# Diffuse fasciitis with restrictive ventilatory impairment and type 2 respiratory failure: a rare case report

**DOI:** 10.1093/rap/rkaf096

**Published:** 2025-08-12

**Authors:** Rinko Katsuda, Akiko Kitagawa, Yoshihiro Seri, Misuzu Fujimori, Tetsuji Kawamura

**Affiliations:** Rheumatology, NHO Himeji Medical Center, Himeji, Japan; Rheumatology, NHO Himeji Medical Center, Himeji, Japan; Respiratory Medicine, NHO Himeji Medical Center, Himeji, Japan; Rheumatology, NHO Himeji Medical Center, Himeji, Japan; Respiratory Medicine, NHO Himeji Medical Center, Himeji, Japan

Key message

- Diffuse fasciitis can be a potential cause of type 2 respiratory failure.

Dear Editor, Diffuse fasciitis (DF) with or without eosinophilia is a rare inflammatory condition characterized by marked fibrosis and thickening of the fascia, often with eosinophilic infiltration [1, 2]. DF is typically associated with limb involvement and may lead to joint motion restriction due to sclerotic changes. Although some cases of trunk involvement have been reported, these largely involve superficial fascial changes without significant respiratory compromise. To our knowledge, no prior reports have described diffuse fibrosis of the trapezius and levator scapulae fascia resulting in restricted thoracic expansion and leading to restrictive ventilatory impairment with type 2 respiratory failure. This case uniquely illustrates how deep fascial involvement of respiratory accessory muscles can compromise pulmonary function, expanding the known clinical spectrum of DF.

A 75-year-old woman presented with type 2 respiratory failure and restrictive ventilatory impairment. Over 5 years she experienced progressive stiffness and pain in the shoulders, neck and back, along with a 10-kg weight loss. Mild peripheral eosinophilia (5.2%, 250/µl) and restricted mobility of the shoulders and neck were noted during earlier evaluations.

Key clinical findings included a height of 145.9 cm, weight 35.8 kg, blood pressure 102/47 mmHg, pulse 67 bpm, temperature 36.8°C and peripheral oxygen saturation 96% on room air. Physical examination revealed extensive subcutaneous induration from the neck to the back, along with stiffness of the sternocleidomastoid muscle, particularly with plate-like sclerotic lesions in the back, along with limited cervical and shoulder joint motion. Raynaud’s phenomenon and other systemic sclerosis–related symptoms, such as finger and facial sclerosis, were absent.

Laboratory tests showed a normal complete blood count except for mild eosinophilia. Autoantibodies, including ANA, anti-RNP and anti-Scl-70, were negative. Serum creatine kinase (47 U/l) and aldolase (2.3 U/l) levels were normal, while IgG (1691 mg/dl) and IgE (278 IU/ml) were elevated. Pulmonary function tests revealed restrictive ventilatory impairment, with a vital capacity (%VC) of 40%. Arterial blood gas analysis indicated hypercapnia, with a partial pressure of carbon dioxide (CO_2_) of 54.5 mmHg, suggestive of CO_2_ retention.

Chest CT performed during both inspiratory and expiratory phases revealed only minimal thoracic expansion, indicating impaired chest wall mechanics. Importantly, no findings suggestive of chronic obstructive pulmonary disease or interstitial lung disease were observed, supporting an extrapulmonary origin of the ventilatory restriction. Contrast-enhanced MRI of the cervical and thoracic regions showed thickening of the trapezius and levator scapulae muscle fascia with abnormal signal intensity on T1 fat-suppressed imaging. Ultrasound demonstrated a thickened and hyperechoic trapezius fascia. A biopsy specimen from the epidermis to the trapezius muscle showed no significant changes in the epidermis, dermis or subcutaneous adipose tissue. However, the fascia of the trapezius muscle exhibited diffuse thickening and sclerotic fibrosis, with perivascular lymphocytic infiltration. No lymphocytic infiltration suggestive of myositis was observed in the trapezius muscle (Fig. 1).

**Figure 1.**
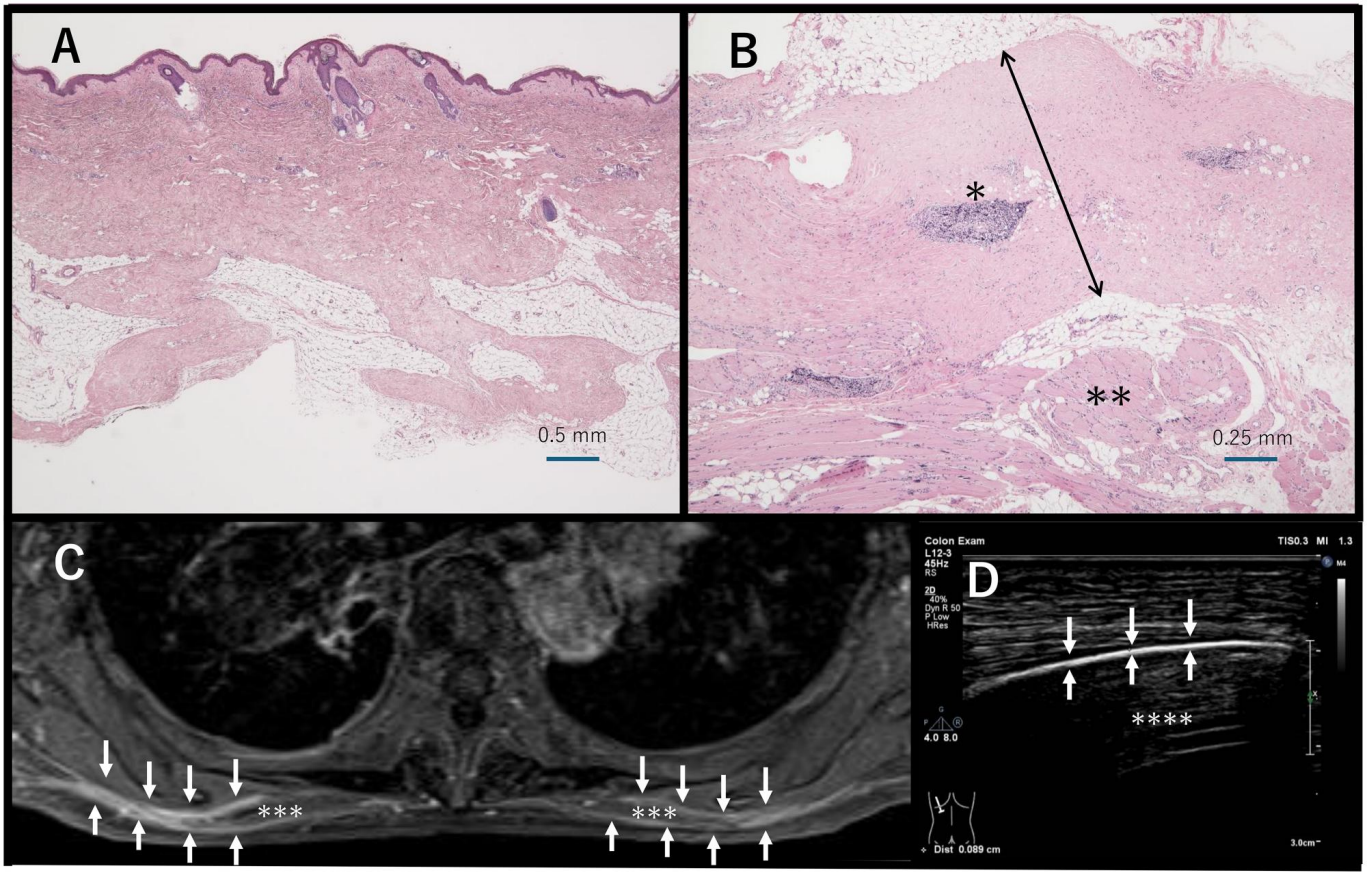
(**A**) Histopathological analysis of the skin biopsy showing no significant changes in the epidermis, dermis or subcutaneous tissues. Scale bar = 0.5 mm. (**B**) Histopathological findings of the trapezius muscle and its fascia. The fascia exhibits diffuse thickening and sclerotic fibrosis (arrow), with lymphocytic infiltration localized around small blood vessels (*). The trapezius muscle shows no lymphocytic infiltration suggestive of myositis (**). Scale bar = 0.25 mm. (**C**) Contrast-enhanced MRI (T1 fat suppression) of the trapezius muscle (***) and its fascia. The fascia reveals diffuse thickening and enhancement (arrow). (**D**) Ultrasound of the trapezius muscle: the fascia (arrow) appears thickened and hyperechoic. A scale is shown on the right margin up to 3 cm

Given the clinical, radiological and histopathological findings, a diagnosis of DF with restrictive ventilatory impairment was made. The patient was treated with systemic corticosteroids (prednisolone 30 mg/day), which were gradually tapered. Within 2 months she exhibited significant improvement in cervical mobility and shoulder function. Contrast-enhanced MRI demonstrated resolution of the abnormal signal in the trapezius muscle fascia. However, pulmonary function tests showed only marginal improvement, with %VC increasing from 40% to 45% and persistent CO_2_ retention. Despite the gradual tapering of prednisolone, no recurrence of fasciitis was observed.

Haematological abnormalities in DF typically include peripheral eosinophilia, which occurs in 63–86% of cases and often appears transiently during the acute phase, decreasing with treatment and correlating with disease activity. In this case, the mild eosinophilia observed in the earlier evaluation indicated disease activity. Regarding the distribution of affected fascia, the patient exhibited extensive subcutaneous induration from the neck to the back, along with stiffness of the sternocleidomastoid muscle. MRI revealed abnormalities in the trapezius muscle but did not detect any abnormalities in the sternocleidomastoid muscle. This suggests the possibility of fascial fibrosis in other muscles that was undetectable by imaging. The primary differential diagnosis is systemic sclerosis, but this patient lacked hallmark features such as finger and facial skin sclerosis, nailfold capillary abnormalities and disease-specific autoantibodies. First-line treatment for DF consists of oral glucocorticoids at a dose of 20–30 mg/day, followed by gradual tapering based on response. The response rate is >90%, but delayed treatment initiation may lead to residual fibrosis and joint contractures. In refractory cases, immunosuppressive agents such as ciclosporin, cyclophosphamide and methotrexate may be considered [3]. In this case, despite a prolonged disease course of 5 years, glucocorticoid therapy improved cervical and shoulder mobility, however, restrictive ventilatory impairment persisted, possibly due to the delayed initiation of treatment.

Type 2 respiratory failure results from impaired alveolar ventilation, leading to CO_2_ retention. It can be caused by a variety of systemic conditions, which are broadly classified into central nervous system disorders, neuromuscular diseases, chest wall and skeletal abnormalities, end-stage respiratory diseases and metabolic disturbances. In neuromuscular disorders such as Guillain–Barré syndrome, amyotrophic lateral sclerosis and inflammatory myopathies, respiratory muscle weakness directly reduces ventilatory capacity. Central causes include brainstem lesions and drug-induced respiratory depression, while chest wall diseases such as ankylosing spondylitis or scoliosis limit thoracic movement. Among systemic fibrosing diseases, systemic sclerosis usually causes type 1 respiratory failure due to interstitial lung disease but may rarely lead to type 2 respiratory failure in end-stage cases with severe pulmonary restriction.

In conclusion, we report a rare case of DF with restrictive ventilatory impairment. Recognizing DF as a rare extrapulmonary cause of type 2 respiratory failure is crucial, especially in patients with progressive thoracic stiffness and unexplained hypercapnia. Prompt diagnosis and treatment may prevent irreversible respiratory compromise and improve functional outcomes.

## Data Availability

The data supporting the findings of this study are included within the manuscript. Additional details are available from the corresponding author upon reasonable request.

## References

[1] Ihn H. Eosinophilic fasciitis: From pathophysiology to treatment. Allergol Int 2019;68:437–9. 10.1016/j.alit.2019.03.001 30910631

[2] Jinnin M , Yamamoto T, Asano Y et al Diagnostic criteria, severity classification and guidelines of eosinophilic fasciitis. J Dermatol 2018;45:881–90. 10.1111/1346-8138.14160 29235676

[3] Berianu F , Cohen MD, Abril A, Ginsburg WW. Eosinophilic fasciitis: clinical characteristics and response to methotrexate. Int J Rheum Dis 2015;18:91–8. 10.1111/1756-185X.12499 25530187

